# Force-velocity coupling limits human adaptation in physical human–robot interaction

**DOI:** 10.1038/s41598-025-34959-4

**Published:** 2026-01-16

**Authors:** Mahdiar Edraki, Hélène Serré, Pauline Maurice, Dagmar Sternad

**Affiliations:** 1 Department of Mechanical and Industrial Engineering, Northeastern University, Boston, USA; 2 Department of Biology, Northeastern University, Boston, USA; 3 Université de Lorraine, CNRS, Inria, LORIA, Nancy, 54000 France; 4 Departments of Electrical and Computer Engineering, and Physics, Institute for Experiential Robotics, Northeastern University, Boston, USA

**Keywords:** Biomedical engineering, Motor control

## Abstract

In physical human–robot interaction, both humans and robots need to adapt to ensure synergetic behavior. This study investigated how humans respond to robots moving with different velocity profiles. In unconstrained human movements, velocity scales with the trajectory’s curvature, i.e., moving fast at linear segments while slowing down at curved segments. Two experiments examined humans tracking a robot that traced an elliptic path with different velocity profiles, while instructed to minimize interaction forces. Results showed involuntary forces were higher when the robot moved with constant velocity or exaggerated the biological velocity-curvature scaling. Specifically, higher angular velocities in the robot were associated with greater tangential and normal forces. Experiment 1 tested whether biomechanical constraints caused these forces by reversing movement direction, but observed differences were small. Experiment 2 explored human adaptation across three practice sessions and found that interaction forces decreased for non-biological profiles only when real-time visual feedback was provided. The force-velocity modulations weakened, indicating that humans learned to predict and compensate for inertial forces. These findings highlight the need to consider human motor limitations and learning processes in physical interaction. The results have practical implications for collaborative and wearable robots where physical contact and coordination between humans and robots are critical.

## Introduction

Studying the determinants that ensure smooth and seamless physical human–robot interaction is critical to integrate robots into human environments^1–3^. This is particularly significant for robots that are designed to work directly with or close to humans, such as collaborative robots, active exoskeletons, or supernumerary limbs. These robotic devices must be effectively coordinated with humans to execute joint tasks successfully, safely and fluently. Optimizing task efficiency, while ensuring human safety, comfort, and fluency is of paramount importance to improve usefulness and acceptance of such devices^4–9^.

Physical human–robot interactions (pHRI) span a spectrum of scenarios, ranging from object handover^10^, to precise placement^11^ and the collaborative transport of an object^12^. In each case, the outcome depends on the synergy between human and robotic movements. But how can this synergy be achieved? Answering this question requires an interdisciplinary approach, combining advancements in robotic control with insights from human movement science to understand and optimize the dynamics of physical collaboration. The dual approach of advancing robotic adaptability and leveraging human motor skill and adaptation forms the foundation for creating robots that can integrate into human-centered environments^13,14^. However, there are still open issues in both robot and human research.

From a robotics perspective, some progress has been made in developing adaptive robot controller that foster intuitive human–robot interactions^15–18^. Key areas of focus have included human intent recognition^19–23^, collision avoidance^24–26^, and optimal action planning^27–30^. These efforts have aimed to create robots capable of adapting to dynamic environments and the nuanced behaviors of their human partners.

Equally important is understanding human preferences and limitations of their adaptive capabilities, as they should inform the design of robotic controllers. Several studies in pHRI research have consistently highlighted human preference for human-like movement patterns in collaborative tasks, such as object transfer^31^, object handover^31^, and exoskeleton-assisted actions^32^. For example,^33^ demonstrated that human reaction times were faster when a robot’s motion adhered to the bell-shaped velocity profile prevalent in unimpeded human reaches, compared to movements with constant velocity that are more typical in robots. Similarly, in a leader-follower pHRI task, our previous research found that participants could better track a robot’s trajectory when it exhibited human motion characteristics, specifically a characteristic scaling of velocity to the trajectory’s curvature, the so-called one-third power law^34,35^.

While programming robots to perform human-like movements may enhance natural interaction, certain task-specific or environmental requirements necessitate non-human-like motion. For example, in applications such as sanding, painting, or precision manufacturing, strictly human-like trajectories may lead to inefficiencies or inconsistencies, such as uneven surface coverage^36^. In these scenarios, human partners must adapt to the robot’s behaviors that are optimized for the task at hand.

Research in human motor control has demonstrated that humans can adapt to novel objects and environments, such as unfamiliar force fields or visuo-motor mappings and objects with unknown internal dynamics^37–39^. Moreover, humans show remarkable flexibility in joint tasks, adjusting their actions based on the partner’s performance, as seen in object handovers and other collaborative tasks^40–42^. A range of studies on human dyadic interactions have demonstrated how humans shape their actions to make them more predictable for their human partner^43^. But can humans adapt to any arbitrary robot motion?

Despite this wealth of adaptation research in human motor control, only few pHRI studies have systematically examined whether or how humans adapt their movements to a robotic partner and how practice affects such adaptation^13^. A notable example is the work by^44^, who demonstrated that over four days of practice, participants improved in a collaborative ball-balancing task with a robot.^45^ also reported human adaptation in a pHRI care-giving task, where the human had to assist a humanoid robot to rise from a sitting position. The study found that humans adapted to the robot’s behavior, regardless of whether or not the robot adapted its behavior to the human. These previous studies focused on human adaptation in scenarios where the robot motion depended on the human behavior^44,46^. This made it difficult to isolate the human’s from the robot’s adaptation. To gain more insights into the specific limitations of the human, our study programmed the robot to traverse a predefined trajectory without regard to the human behavior.

The specific factors limiting human adaptation to robots, with or without *human-like* movement profiles, remain unresolved. Previous work from our lab examined humans tracking a robot moving around an elliptical path with different velocity profiles^34,35^. We reported significantly higher undesired interaction forces when the robot’s velocity did not adhere to the preferred one-third power law, but the forces slightly decreased with practice. While visual feedback of the force improved the interaction with the robot, humans continued to exert unintended forces. Similar observations were made by^47^ when participants were also asked to simultaneously apply a constant tangential force to the robot. While those studies suggested inherent limitations in human motor control or biomechanics, their experiments had some design limitations and were only first probes into this multi-faceted question about the determinants responsible for undesired and unintentional forces.

To address these open issues, the present study designed a pHRI experiment that aimed to isolate specific parameters that might pose challenges to human adaptation. Two experiments tested three different velocity profiles around an elliptic path in the horizontal plane and compared forces that humans unintentionally exerted against the robot’s motion. A new set of analyses scrutinized how forces depended on variations of the robot’s instantaneous velocity along the path. Experiment 1 first examined whether biomechanical factors, introduced by different movement directions around the ellipse, affected the applied forces. Experiment 2 explored whether practice, extended over several days, could overcome the limitation identified in Experiment 1, and whether real-time feedback could help. Detailed analyses of the continuous forces highlighted how the patterns decreased with practice.

## Methods

### Participants

The two experiments in this study involved a total of 59 healthy college students, 32 females and 27 males, aged between 18 and 35 years. Experiment 1 involved 18 participants and Experiment 2 involved 41 participants. Participants were recruited from a convenient sample of local college students. They had little to no experience in robotics, and were not familiar with human–robot interaction. Participants had no reported biomechanical issues in their upper limbs, and were unaware of the purpose of the study. All participants were right-handed and used their dominant right hand to perform the task in both experiments.

The experimental procedure was approved by the Institutional Review Board of Northeastern University (#10-06-19) in accordance with the ethical requirements of the university and with the Helsinki Declaration and its later amendments. All participants signed an informed consent form prior to participation.

### Experimental apparatus

**Fig. 1:**
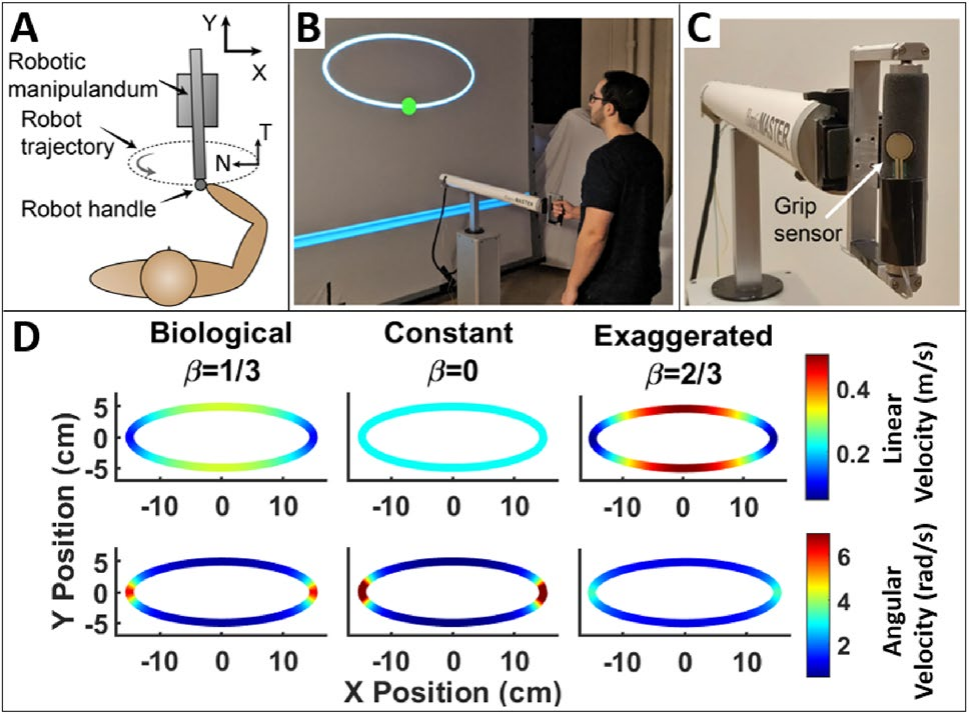
(**A**) Top-down view of the experimental setup showing the relative positions of the robot, human, and the elliptical path. X and Y show the positive axes for the robot’s end-effector movement. Positive tangential (T) forces point in the direction of motion and positive normal (N) forces point towards the center of the ellipse. (**B**) A participant performs the task while observing the screen displaying the ellipse and a cursor representing the human–robot motion along the path. Cursor color indicated force magnitude: green within the threshold, red exceeding it. (**C**) Close-up of the robot handle with a force sensor ensuring participant engagement. (**D**) Linear and angular velocity profiles of the biological, constant, and exaggerated conditions, illustrating velocity changes around curved and straight sections of the ellipse.

The robot used in both experiments was the HapticMaster, a 3-degree-of-freedom (DOF) robotic manipulandum (Motek Medical, The Netherlands)^48^, depicted in Fig. 1A C. The robot was programmed to have its end-effector trace an elliptical path in a horizontal plane; the ellipse had a major axis of 30 cm and a minor axis of 10 cm. For this horizontal motion, only 2 joints of the robot were required –and therefore active– to trace the desired 2-DOF trajectory; the third joint was locked throughout the task, thereby excluding any redundancy.

Figure 1A illustrates the participant’s position relative to the robot and the elliptic path. The robot was positioned in front of a large backprojection screen, that showed the ellipse in the frontal plane with a circular cursor representing the robot’s end-effector position traversing around the ellipse in real time (Fig. 1B).

Participants interacted with the robot via a handle attached to the end-effector (Fig. 1C). The handle incorporated a vertical bar that could freely rotate around its vertical axis, thereby mechanically decoupling the orientation of the robot’s end-effector from the orientation of the participant’s wrists. To ensure that participants firmly held the robot handle and were actively engaged in the task, a force-sensitive resistor (FSR) was mounted on the handle to monitor their grip force.

The robot was controlled using the admittance controller provided by the manufacturer through the manufacturer’s C++ API^48^. Using this built-in admittance controller, the robot end-effector motion emulated the behavior of a virtual mass submitted to both the force applied by the participant on the robot handle and the force resulting from a virtual spring-damper system, whose extremity tracked a reference trajectory. The reference trajectory inputted to the robot controller corresponded to the elliptical path traced according to the different velocity profiles (see next section). The reference position was updated at 120 Hz. The virtual mass was set to $2~\textrm{kg}$, as per the manufacturer’s recommendation. The virtual stiffness and damping parameters were set to $10000~\mathrm {N/m}$ and $2~\mathrm {N\,s/m}$, respectively. These values were tuned experimentally to ensure the robot end-effector accurately tracked the reference trajectory, even when participants applied force on the handle during the task (the tracking error remained below 0.4 cm for the range of forces typically applied by participants in the task). The high stiffness value resulted in a stiff behavior of the robot, very similar to position control.

Interaction forces from the participants on the robot were measured using the HapticMaster’s built-in 3-axis force sensor located at the end-effector, provided by the manufacturer^48^. This sensor measures the 3D components of the force, but did not include torque measurements. End-effector position and interaction forces were recorded at 120 Hz for subsequent analysis.

### Robot velocity profiles

Previous human movement studies demonstrated that endpoint trajectories follow the so-called *one-third power law*, which describes a systematic relation between the linear velocity and the radius of curvature of the associated path^49–51^. For trajectories without inflection points or entirely linear segments, this relation can be expressed mathematically as:

1 $$v(t) = K r(t)^\beta$$

*v* represents the linear hand velocity, *r* denotes the radius of curvature of the path, and *K* is a gain factor on the velocity of the trajectory. For human trajectories, $\beta$ was found to be close to 1/3. This equation can also be formulated in terms of angular velocity and curvature with a corresponding exponent of 2/3. Hence, this observation has also been referred to as the “two-third power law”. This relation effectively captures how movement decelerates in sections of the path with higher curvature and accelerates in straighter segments, doing so in a consistent and predictable manner. This one-third power law was first identified in cursive hand writing, but then generalized to a large range of hand and even locomotory movements. This power law has been used here to generate “biological” movement patterns, with deviations from this law considered as “non-biological”^52^.

To test whether humans can adapt to non-biological robot trajectories, this study designed three different velocity profiles for the robot’s endpoint trajectories. By adjusting the exponent $\beta$ in equation (1), three distinct velocity profiles were generated: *biological* ($\beta = 1/3$), *constant* ($\beta = 0$), and *exaggerated* ($\beta = 2/3$) (Fig. 1D). In the *biological* profile, the robot followed the ellipse with its velocity slowing down in curved sections and speeding up in straighter ones, mimicking natural human movement. In contrast, the *constant* profile imposed a uniform linear velocity on the robot, akin to common control strategies in robotic motion. The *exaggerated* profile amplified the natural variations of the *biological* profile, causing the robot to move even more slowly in curves and more quickly in straight segments of the ellipse. The gain factor *K* was adjusted for each velocity condition to impose a 3-second period while tracing the ellipse for all three conditions.

### Experimental procedure and instructions

Prior to both experiments, participants were positioned in front of the robot facing a large backprojection screen (Fig. 1B). The experimenter individually adjusted the height of the robot such that each participant’s forearm was nearly horizontal when holding the robot’s end-effector. Participants were allowed to find a comfortable distance from the robot and were told not to move between trials.

Participants were instructed to firmly grip the robot handle with their dominant right hand and actively follow the robot’s movements to neither drag, nor push against the robot. To ensure that participants maintained a firm grip on the handle and fully engaged in the task, a force-sensitive resistor (FSR) was mounted on the handle to estimate grip force (Fig. 1C). If the participant’s grip force fell below a specified threshold, the sensor triggered an auditory cue (buzzer). This was necessary to prevent participants from reducing their force on the handle and simply loosen their grip during the experiments, which could have falsely indicated low force exertion. The trigger threshold was empirically calibrated to be below their natural grip force, so that it did not interfere with preferred task performance. The FSR was used solely for monitoring active participation and in the data pre-processing phase to exclude trials in which participants did not sufficiently grip the handle. After that, the FSR data were not included in any further data analyses.

### Experimental questions and hypotheses

The overarching aim of the two experiments was to test whether humans were able to track different velocity profiles without exerting undesired forces against the robot. The main hypothesis was that humans exert higher forces against non-biological robot motions than against the biological profile. Experiment 1 first asked whether biomechanical factors contributed to the exerted forces or whether undesired forces reflected a control limitation. By comparing clockwise and counterclockwise elliptical motions, it examined whether biomechanical asymmetries in muscle function and inertial dynamics influenced the results. The hypothesis was that the primary factor eliciting undesired forces in human performance was the velocity profile, i.e., limitations in control, and not the direction of motion, i.e., muscle and joint biomechanics. This question was further detailed by examining how forces continuously varied with the specific velocity along the ellipse. Experiment 2 tested whether humans could learn to reduce the undesired forces over extended practice and by being given augmented visual feedback. We hypothesized that the added visual feedback would enable subjects to adapt to non-biological patterns. We also examined how such improvements depended on the changing velocity around the ellipse.

**Fig. 2:**
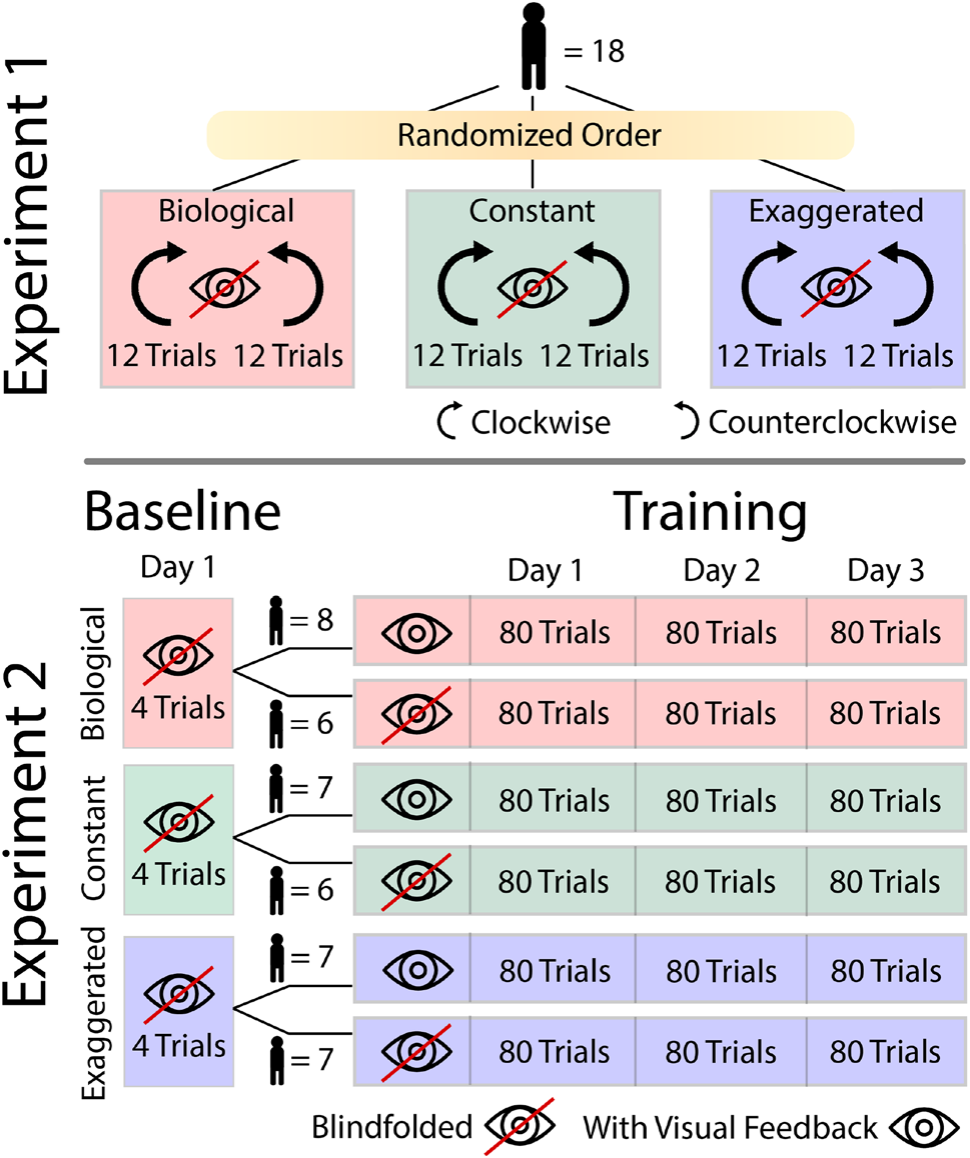
Experimental design. In Experiment 1, each participant performed the three velocity profiles in clockwise (CW) and counterclockwise (CCW) direction, in 2 blocks of 12 trials. Experiment 2 tested the same velocity profiles, with or without visual feedback, across six participant groups (group sizes denoted by n). Each group practiced for 3 days, completing 80 trials daily, totaling 1440 elliptical movements per group. Day 1 began with 4 blind-folded baseline trials in each group’s assigned velocity condition.

#### Conditions in Experiment 1

In this study all 18 participants performed three velocity profiles, *biological*, *constant*, and *exaggerated*; each profile was performed in two directions *clockwise* (CW) and *counterclockwise* (CCW). As overviewed in Fig. 2, each velocity condition comprised 12 trials per direction, grouped into blocks, with the order of the direction blocks counterbalanced across participants. The three velocity conditions were presented in random order for participants. Each trial comprised 4 ellipses, each of 3 s duration (12 s total). After each block of 12 trials, there was a break of 2-3 min to mitigate fatigue, resulting in a total of 30 min for the experimental session. Participants were blind-folded throughout the experiments, receiving no visual information about the robot’s motion.

#### Conditions in Experiment 2

Experiment 2 investigated whether participants could improve their performance in the three velocity profiles with extended practice and whether augmented visual feedback positively affected performance improvement (Fig. 2). Six groups of participants (6 to 8 participants each) practiced one of the three velocity conditions, either blind-folded or with visual feedback. Regardless of the feedback condition, all participants experienced haptic and proprioceptive feedback through their interaction with the robot handle. Each group performed their assigned condition over 3 sessions on 3 consecutive days. Each session contained 80 trials (240 trials per day), and lasted approximately 1 hour.

The robot traced the elliptical path 6 times per trial always in the CCW direction, with each trial lasting 18 s. To prevent fatigue, participants were given 5 s rest between trials. Additionally, participants received a 2-3 min rest after completing 10 trials. As in Experiment 1, prior to the first practice session, all participants performed 4 trials in their assigned velocity profile with no visual feedback. These trials established *baseline* performance that was used to determine the thresholds for the visual feedback.

To assess whether visual feedback aided learning, real-time feedback was provided to participants in three of the six groups (Fig. 2). This online feedback was shown on the projection screen by a circular cursor tracing the ellipse as the robot moved along its predefined elliptical path (Fig. 1B). The color of the cursor could change between green (good) and red (bad) to signal the force applied to the handle. Real-time force was quantified by the root mean square (RMS) of the magnitude of force that the participant exerted, calculated in a sliding window of 80 ms. The initial threshold for the color change was based on the participant’s average force of their 4 *baseline* trials: the cursor was green, if the error was less that half of their *baseline* force, and it was red, if it rose above twice of their *baseline* force. The threshold was progressively updated every 40 trials to uphold the challenge to reduce force. Participants who received visual feedback were instructed to aim for a green cursor.

### Data preprocessing

Before the main data analyses, grip forces of all individual trials were inspected to ensure adequate grip was maintained throughout the task. Trials were excluded if the buzzer was triggered for more than 10% of the trial duration (i.e., >1.2 s out of 12 s for Experiment 1 and >1.8 s out of 18 s for Experiment 2). This led to the exclusion of approximately 3% of trials, primarily from two participants in Experiment 2. These participants, one from the *biological, no-FB* group and one from the *constant, no-FB* group, were removed from the study. This procedure eliminated the possibility that low forces were simply due to an insufficient grip force.

The force sensor on the robot recorded participant forces in the x- and y-directions of the horizontal plane. These measures were converted to force magnitude and its component tangential and normal forces at each point along the elliptical path. Data were low-pass filtered with a 4th-order Butterworth filter with a 6 Hz cutoff to retain over 90% of the original signal’s power. Transient effects were minimized by excluding the first and last ellipse of each trial in both experiments.

### Performance evaluation-dependent measures

#### Root mean square force

To address the main hypotheses of both experiments and assess participants’ exerted force across different velocity profiles, including direction conditions and practice days, the forces measured at the robot handle along the ellipse were summarized using the root mean square (RMS) of the forces per trial. In addition to the overall magnitude, the forces were decomposed into their two orthogonal components: *tangential* (parallel to the path) and *normal* (perpendicular to the path). This resulted in three dependent measures per trial: RMS force magnitude, RMS tangential force, and RMS normal force.

**Fig. 3:**
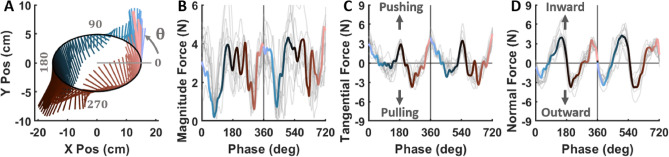
Interaction forces (direction and magnitude) exerted by a representative participant on the robot following the *biological* velocity profile in the *CCW* direction. Force vectors show the applied forces across two ellipses in a representative trial. The definition of phase is displayed in panel **A**, **B**, **C**, and **D** display the phase progression of the force magnitude, tangential force, and normal force, respectively. Gray lines represent all trials, and the colored lines show the same trial as in **A**. Vertical lines mark the completion of the first ellipse.

Figure 3 displays the interaction forces exerted by a representative participant in Experiment 1 during the *biological* velocity profile in the *counterclockwise* direction. Panel A shows a top-down view of the elliptical path in the x-y plane with forces overlaid from two successive ellipses. The color-code represents the temporal evolution across 360 deg phases. Despite the instructions to minimize force, the participant consistently exerted significant non-zero forces.

Panels B, C and D illustrate the evolution of force magnitude, and the tangential and normal force components over the same two cycles in the *CCW* condition. The solid colored lines indicate the force of the two successive ellipses, while the gray lines represent two cycles from all 12 trials. For both *CW* and *CCW* directions, positive tangential forces reflect pushing against the robot, while negative values indicate lagging behind it. Positive normal forces represent an inward push towards the center of the ellipse, while negative forces indicate an outward push. Despite some variations between trials, the overall force pattern remained quite reproducible, both across cycles within a trial and between trials.

#### Median forces along the ellipse

In a second step, this study sought to uncover what aspects of the elliptic trajectory gave rise to the undesired forces. To address this question, the median tangential and normal forces were calculated across trials at each phase along the ellipse, offering a spatially resolved summary of the applied forces.

In Experiment 1, the median forces were computed across 12 trials (2 ellipses each), producing one median force trajectory per experimental condition for each participant. In Experiment 2, median forces were calculated across 80 trials per day (4 ellipses per trial), providing a summary force pattern for each participant per day.

**Fig. 4:**
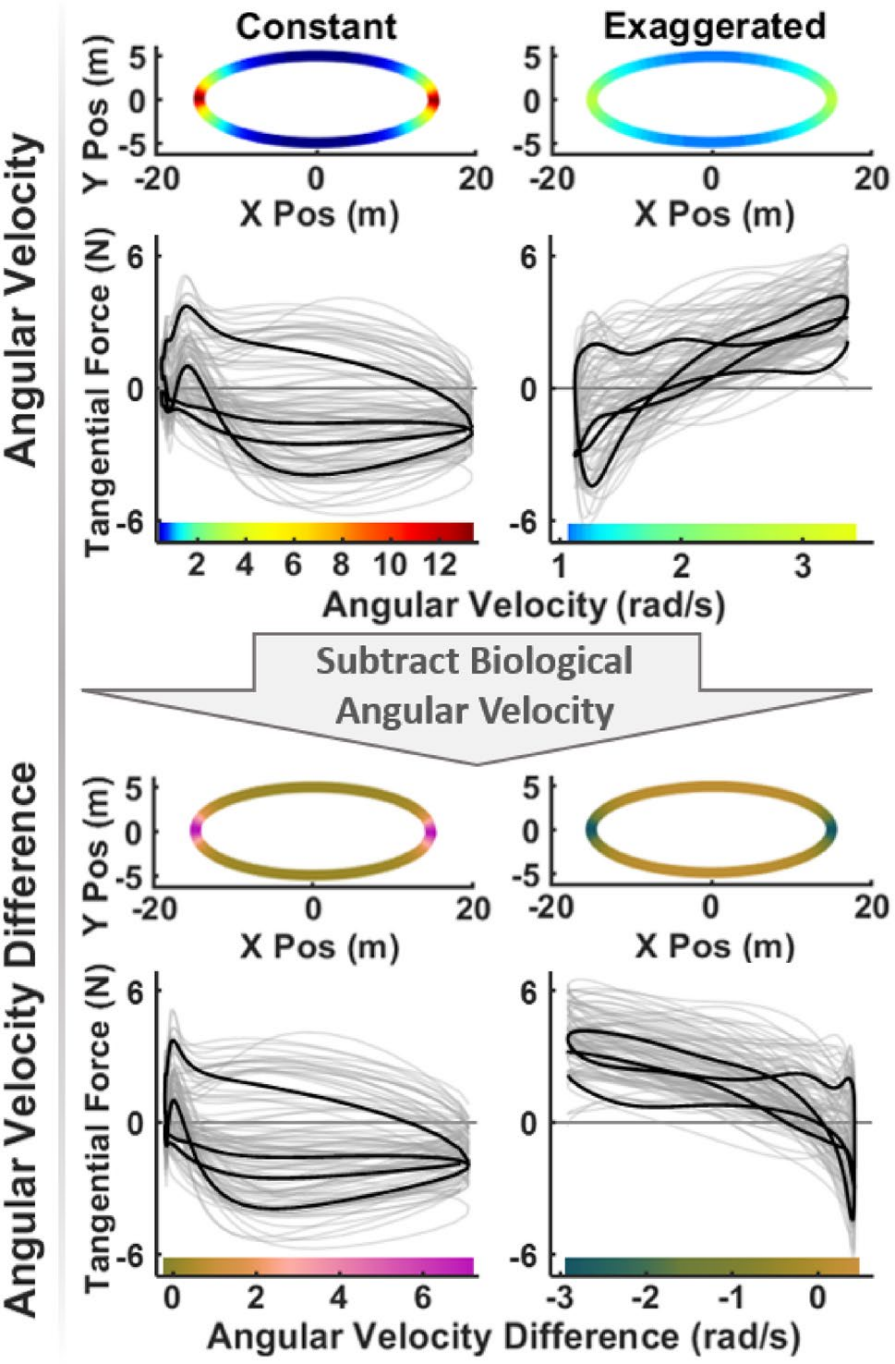
Velocity profiles of the robot along the ellipse and regressions of applied tangential forces against angular velocity (top) and angular velocity difference (bottom) for the two non-biological velocity profiles. The ellipses show the angular velocities and the angular velocity difference; the latter was calculated by subtracting the biological profile’s angular velocity from the constant and exaggerated profiles (visualized with updated color codes). The gray lines show the tangential force applied in 12 trials (24 ellipses) at each spatial point along the ellipse by two representative participants in the *CCW* direction. The solid lines highlight forces applied across a representative ellipse

To explore the relation between the applied forces and the robot’s motion, key kinematic features such as path curvature, linear velocity, and angular velocity were considered as possible causes for the undesired forces. However, path curvature was ruled out as this was the same for all velocity conditions and therefore could not distinguish between velocity profiles. Linear velocity of the robot trajectory could also be ruled out as a candidate, as this velocity was constant in the *constant* velocity condition, yet clear force modulations were visible across the ellipse (see results). Hence, regression analyses focused on how forces depended on angular velocity of the robot trajectory. Figure 4 demonstrates the steps for this analysis. At the top, the ellipses illustrate the angular velocity of the two non-biological profiles; below the tangential force applied in all 12 trials were plotted against instantaneous angular velocity for the *constant* and *exaggerated* velocity conditions. The solid black line represents the forces applied in a representative trial.

Given our main hypothesis that deviations from the human-preferred velocity profile elicits additional forces, we also computed the difference between the biological angular velocity and the corresponding values in the constant and exaggerated profiles at each spatial point along the ellipse. The bottom of Fig. 4 illustrates this difference of angular velocity around the ellipse (with a new color code) and also the corresponding linear regressions of tangential force against this angular velocity difference. As above, the solid black line represents the same representative trial.

### Statistical analyses

The two experiments used within and between subject factors. Especially for the mixed design in Experiment 2, it was important to make sure that the number of participants was sufficient to ensure statistical power. The sample sizes were determined by power analyses aiming for 80% power, using pilot data as well as previous data from our group^34^. For the within-subject design of Experiment 1, the sample size was determined using the software G*Power. In Experiment 2, determining the required sample size relied on the three-way interaction (feedback velocity profile day) which included within and between factors. Because standard tools such as G*Power could not handle this three-way mixed design, we performed the power analysis with our own simulations. This involved the following steps: generate 1000 different datasets using the effect sizes from pilot or prior studies, analyze each dataset with the linear mixed model, obtain the p-value related to the three-way interaction, compute the proportion of significant p-values for the interaction in the 1000 simulation runs. We performed these calculations for an incrementally increasing number of participants to determine the sample size needed to reach 80%. These simulations used pilot data as well as previous data from a previous study by our group^34^.

In both experiments linear mixed-effects models were used to test the hypotheses. These regression models included fixed factors, allowing random intercept and random slopes for each fixed factor; participants were included as a random factor to account for inter-participant variability. The significance of the main effects and interactions was assessed through likelihood ratio tests (LRT). Post-hoc comparisons between levels of categorical variables were evaluated at a 95% confidence level, using Tukey’s HSD corrections^53^.

For the statistical comparisons of the RMS force magnitude, RMS tangential, and RMS normal force, a log transformation was applied to the force data prior to analysis. This step addressed the inherent positive skewness of the three RMS variables and ensured a more normal data distribution, improving the robustness of the statistical tests. All analyses were conducted in R (version 4.3.2) using the *lme* function from the *nlme* package for mixed-effects modeling^54^.

#### Experiment 1

The first set of analyses examined how the RMS force values differed between the velocity profiles and the two directions (including magnitude, tangential and normal forces). To examine their effects on force, the model included the fixed factors *velocity profile* with three levels (*biological*, *constant*, and *exaggerated*), and movement direction with two levels (*CW* and *CCW*), and their interaction. Trial number (1 to 72) was also included as a fixed factor to account for potential improvement or fatigue within the session.

The second set of analyses assessed whether the continuous force depended on the angular velocity and/or angular velocity difference along the ellipse. A linear mixed model was applied to the median of the tangential and normal forces across trials at each point on the ellipse, with fixed effects including velocity profile, movement direction, angular velocity (or angular velocity difference), and their interactions.

#### Experiment 2

To evaluate performance improvements in the RMS forces across practice days and feedback conditions, the linear mixed model included fixed effects for velocity profile, visual feedback (*with-FB* vs. *no-FB*), Day (*1*, *2*, and *3*), and their interaction. Trial number (1 to 240) was also included as a fixed factor to account for within-day learning effects.

For conditions showing significant improvement through practice, further analysis investigated how the decrease in RMS force was linked to the force-velocity coupling. The regression model was applied to the continuous trajectories of the median tangential and normal forces across 80 trials per day against angular velocity difference, and incorporated fixed effects for velocity profile, angular velocity difference, day (*1* and *3*), and their interactions.

## Results

Two experiments tested how different velocity profiles of the robot elicited undesired forces exerted by participants. Experiment 1 explored whether movement direction affected force exertion, Experiment 2 examined whether extended practice with real-time visual feedback could mitigate any undesired forces. Additionally, detailed analyses of continuous forces examined how deviations from biological velocity profiles contributed to excessive forces, providing deeper insights into the dynamics of human–robot interaction.

**Fig. 5:**
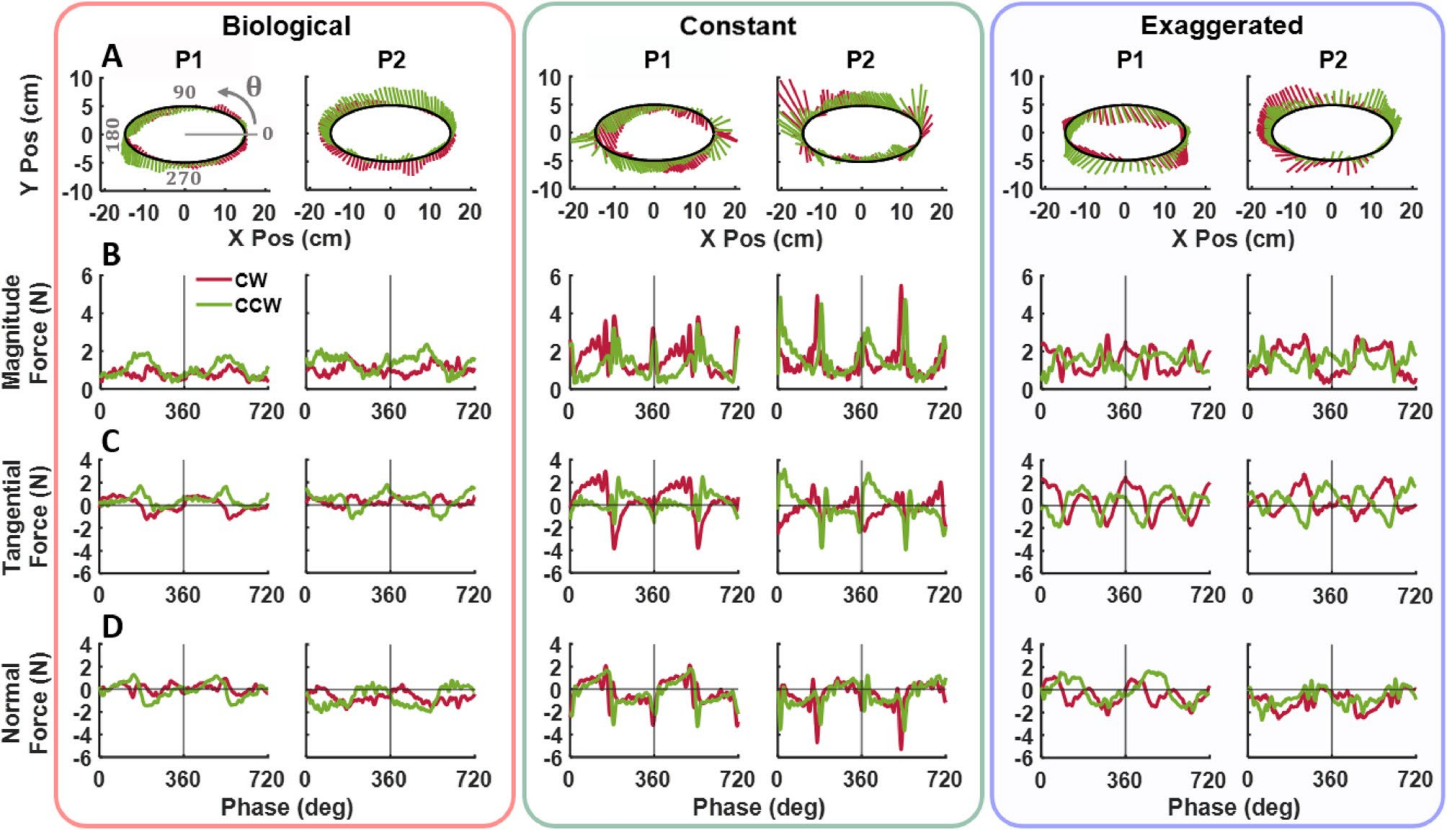
Interaction forces (direction and magnitude) exerted on the robot across the 3 velocity profiles in both *CW* (red) and *CCW* (green) directions from two representative participants, P1 and P2, in Experiment 1. (**A**) The vectors represent the median force applied across all 12 trials. (**B**) Median magnitude of force applied across 2 ellipses as a function of phase, $\theta$, defined in (**A**). The vertical lines indicate traversal of one ellipse. (**C**) Median tangential forces across 2 ellipses. (**D**) Median normal forces across 2 ellipses.

### Experiment 1

This experiment tested the hypothesis that non-biological velocity profiles elicit higher undesired forces compared to biological velocities and that these effects are unaffected by the direction of rotation around the ellipse. If there was a difference between the *CW* and *CCW* rotations, this would indicate that additional biomechanical factors needed to be considered.

#### Performance in different velocity profiles and directions

Figure 5 displays the median applied force for two participants across the 12 trials in the three velocity profiles, in both *CW* and *CCW* directions. Panel A shows the path-dependent forces around the ellipse, panel B shows the phase progression of the force magnitude, and panels C and D its two components, tangential and normal force, over two elliptic cycles. The two colors indicate the two directions. These exemplary data visualize that the three different velocity profiles elicited different force patterns, while the directions of motion did not generate visibly different force patterns. The modulations of the forces differed between the two participants in all conditions. Despite this seemingly unsystematic behavior between subjects, the force patterns repeated themselves across the cycles within each subject. Even though relatively small, the forces in the *biological* condition appeared to exhibit the lowest values, regardless of movement direction (*CW* or *CCW*). A linear mixed model evaluated these observations statistically.

**Fig. 6:**
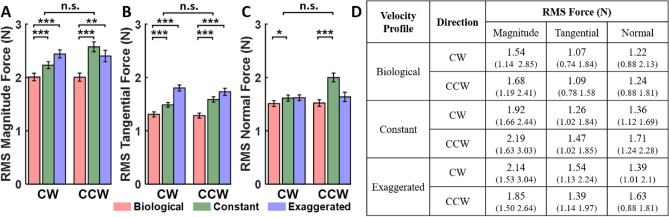
Average root mean squared (RMS) forces applied by participants across the three velocity profiles both in *CW* and *CCW* directions. Error bars indicate standard error. (**A**) Force magnitude. (**B**) Tangential force. (**C**) Normal force. Asteriskses signify significant differences in pairwise posthoc comparisons ($$*$$: $$p < 0.05$$, $$**$$: $$p < 0.01$$, $$***$$: $$p < 0.001$$). (**D**): Median and interquartile range (in parentheses) of RMS force for each condition.

Figure 6 summarizes the statistical results, showing average RMS values for force magnitude, tangential, and normal components, separated by direction. Participants exerted significantly lower forces across all components in the *biological* profile compared to the *constant* and *exaggerated* profiles. Pairwise comparisons showed significant differences between the *biological* and *non-biological* profiles for all force components, except for normal force between *biological* and *exaggerated*. Movement direction (*CW* and *CCW*) showed no significant main effect. However, an interaction between *velocity profile* and *direction* revealed that in the *constant* profile, forces (magnitude and normal components) were higher in the *CCW* direction compared to *CW*. The median and interquartile range of the applied forces are listed in the adjacent table. These results overall supported the main hypothesis that humans can better follow a robot that exhibits human-like movements. This behavior was not influenced by the movement direction of the robot, supporting the second hypothesis. Detailed statistical results can be found in Table S1 in the Supplementary Materials.

**Fig. 7:**
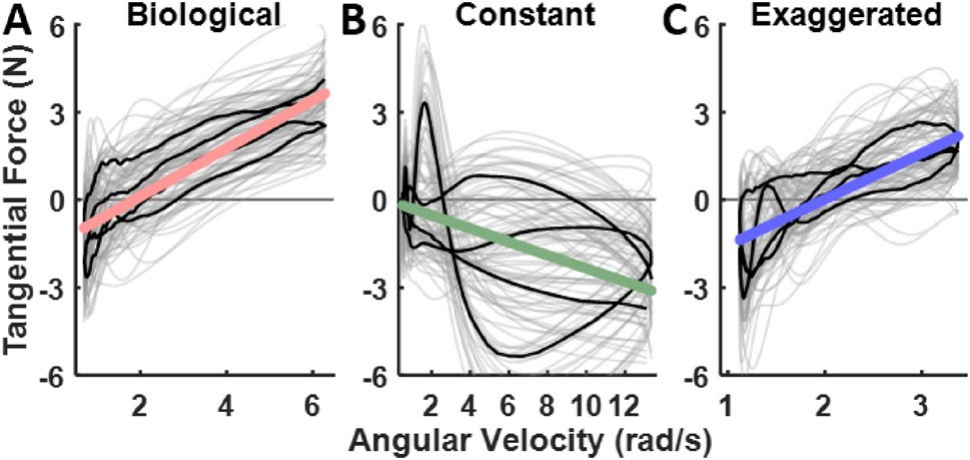
Applied tangential force plotted against angular velocity at each sample time for all 12 trials (gray lines) for a representative participant in the *biological* (**A**), *constant* (**B**), and *exaggerated* (**C**) velocity profiles in the *CCW* direction in Experiment 1. The median of the force is shown by the black trace with the linear regression (colored lines) of the median of force versus angular velocity for the same participant. Note that the x-axis scale is different for each velocity profile.

#### Force-velocity coupling

Figure 5 illustrates how the continuous interaction forces were modulated along the ellipse, which was different for the different velocity profiles. What features in the robot motion between profiles elicited these force modulations? As curvature was identical for all three velocity profiles, curvature could not account for the different patterns. A second candidate could be linear velocity, but the unchanging linear velocity in the *constant* condition still elicited systematic force modulations along the ellipse. Hence, the third remaining candidate was angular velocity of the robot motion.

To investigate this, we regressed the tangential and normal force trajectories against the robot’s instantaneous angular velocity, applied to all participants’ data within each condition. This analysis focused on the two force components (tangential and normal) enabling a more thorough investigation of how and why robot motion was resisted by the human operator. Figure 7 illustrates this analysis with representative results for the 3 velocity conditions. The colored lines show the linear regressions for these selected conditions (tangential force in the *CCW* direction), serving as a visual demonstration of how the force scaled with angular velocity.

**Fig. 8:**
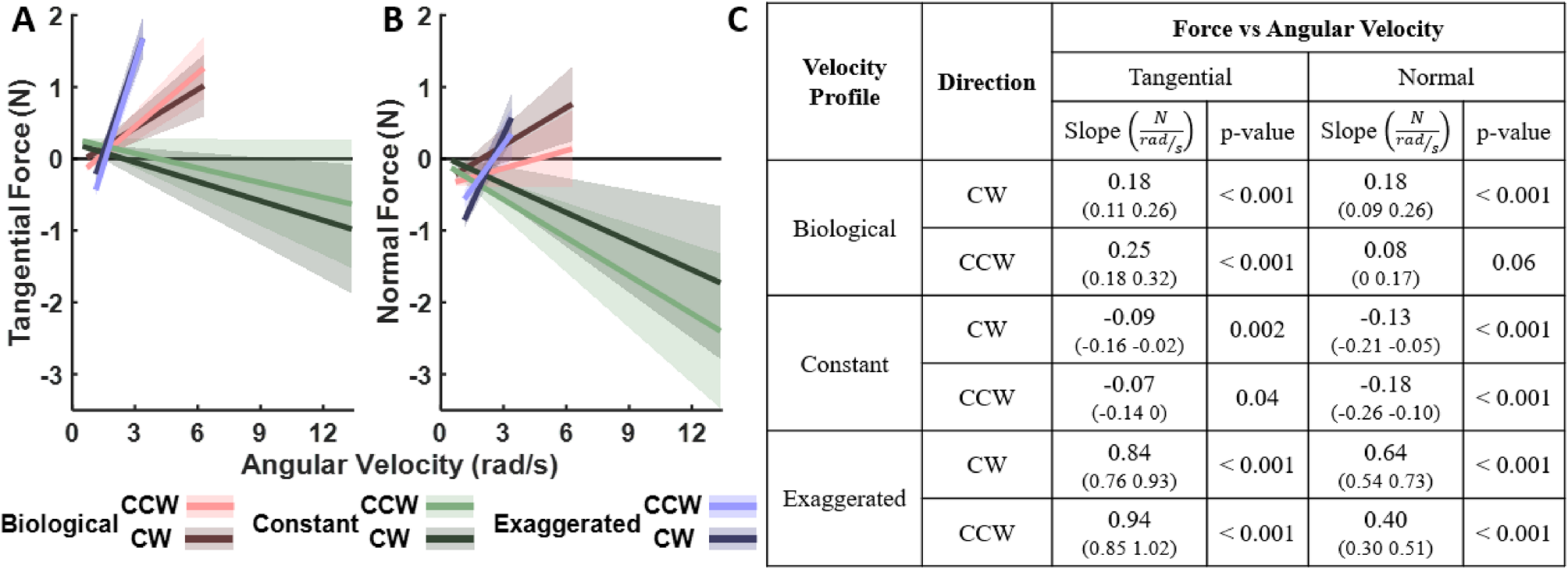
Results of the statistical regression analysis that tested the relation between the forces on angular velocity in Experiment 1. The plots on the left show the mean slopes and intercepts and their corresponding 95% confidence intervals for tangential (**A**) and normal (**B**) forces, respectively. Velocity conditions and motion directions are represented by different colors ,whilethetwomotion directions are distinguished by different shades. The table on the right (**C**) presents the slopes and their corresponding 95% confidence intervals (in parentheses) as well as p-values for post-hoc comparisons of interest, testing specifically whether the slopes differed significantly from zero. The complete table is included in the Supplementary Material (Table S2).

Figure 8 summarizes the statistical results, both graphically and numerically in the table. It reveals significant linear relations between the applied forces and the robot’s angular velocity in all three velocity profiles, in both *CW* and *CCW* directions. For the tangential forces, the negative slopes in the *constant* velocity profile signified increased lagging or resistance against the robot as angular velocity increased. Conversely, the *biological* and *exaggerated* profiles exhibited positive slopes, indicating that participants pushed against the robot more during faster traversals. Inspection of the normal forces shows that participants applied stronger negative (outward) forces in the *constant* condition as angular velocity increased, whereas in the *biological* and *exaggerated* profiles, participants applied stronger positive (inward) forces. These trends were indifferent to movement directions. The statistical results are listed in the adjacent table and also in more details in the Supplementary Material.

**Fig. 9:**
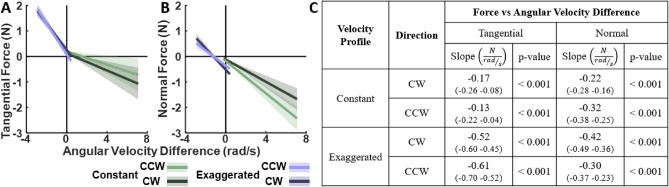
Results of the statistical regression analysis that tested the relation between the forces on the difference between angular velocity in the biological condition and the constant and exaggerated conditions respectively in Experiment 1. The plots on the left show the mean slopes and intercepts and their corresponding 95% confidence intervals for tangential (**A**) and normal (**B**) forces, respectively. Velocity conditions and the two motion directions are represented by different colors. The table on the right (**C**) presents the slopes and their corresponding 95% confidence intervals (in parentheses) as well as p-values for post-hoc comparisons of interest, testing specifically whether the slopes differed significantly from zero. The complete table is included in the Supplementary Material (Table S3).

Building on the results that the RMS forces against the robot were lower for biological velocities, we also calculated the angular velocity difference at every spatial point along the ellipse. Regressions of the *constant* and *exaggerated* profiles against this angular velocity difference sought to identify whether there were even clearer dependencies than for angular velocity alone. The results, overviewed in Fig. 9, reveal that angular velocity difference significantly predicted the forces with clear negative slopes in both *constant* and *exaggerated* conditions. These relations were again independent of the movement direction. This indicated that indeed the deviations from biological angular velocity were a primary driver for the observed forces.

### Experiment 2

Experiment 2 investigated whether humans could learn to make their interaction with a robot less effortful, moving with the same three velocity profiles. Visual feedback was introduced as a new element to probe whether it could help overcome the undesired effects. The main hypothesis was that humans could adapt to non-biological profiles and reduce their applied forces, especially when visual feedback was provided. Since movement direction did not significantly influence the applied force in Experiment 1, this experiment was collected with only one rotation direction (*CCW*). To prevent carry-over effects between different velocity and feedback conditions, each of the six experimental conditions was performed by a separate group of participants.

#### Performance changes with practice

**Fig. 10:**
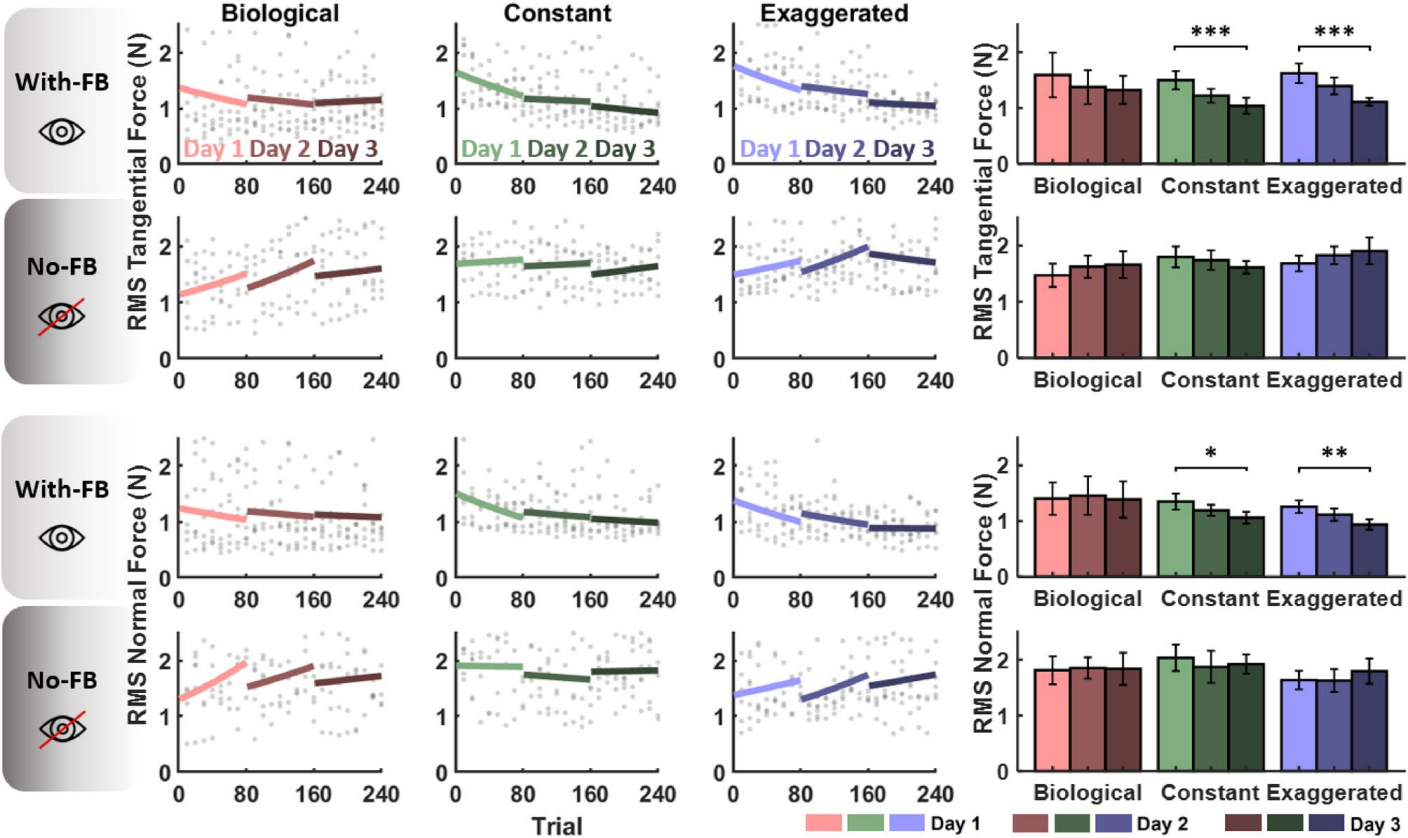
RMS tangential and normal forces across 3 practice days in all six experimental conditions. The left plots display individual participant data as gray dots (averaged across 10 trials), with regression lines indicating performance trends within each day. Colors represent the velocity profiles, and darker shades correspond to later practice days. The right plots show all participants’ mean RMS forces for each day, with error bars indicating standard error. Statistically significant reductions in applied force across days are marked with asterisks, highlighting improvements through practice ($$*$$: $$p < 0.05$$, $$**$$: $$p < 0.01$$, $$***$$: $$p < 0.001$$).

The first analysis examined overall performance across trials and days using the RMS values of the tangential and normal components of applied force, calculated for each of the 240 trials. Force magnitude was no longer included. Figure 10 displays the RMS tangential and normal forces across the three practice days for all six participant groups (three velocity profiles, with and without feedback). For visualization, the RMS force for each participant, shown as gray dots, was averaged over sets of 10 trials. Despite the substantial variability in the force time profiles of the individual subjects (Fig. 5 above), a clear pattern emerged in the collective behavior: For both tangential and normal forces, a decline in force is visible in the *constant* and *exaggerated* profiles when feedback was provided.

To buttress this observation statistically, a linear mixed model compared RMS forces across velocity profiles, feedback conditions, days, and trials; separate tests were performed for tangential and normal forces. The regression lines across trials for each day are shown in the left panels of Fig. 10. The bar charts on the right summarize the mean RMS forces of the three velocity conditions across days. Comparing performance changes across trials within each day, a significant reduction of tangential force was observed on Day 1 for the three participant groups who received visual feedback. For normal force, only participants in the *constant* condition who received visual feedback showed a significant change across Day 1.

Looking at the changes from Day 1 to Day 3 participants with visual feedback showed significant reductions in force in the *constant* and *exaggerated* profiles. The asterisks above the bar charts indicate significance. The effect sizes for the change from Day 1 to Day 3 were 0.625 and 0.25 for constant and exaggerated respectively, while only 0.313 and 0.11 for normal force. In contrast, no significant changes were observed in the *biological* profile across days, not even when visual feedback was provided. Interestingly, no significant reduction in applied force was observed when participants did not receive visual feedback. This absence of improvement was seen in both normal and tangential forces. Comprehensive statistical results are available in Table S4 in the Supplementary Materials. These results support the main hypothesis of Experiment 2 that subjects could adapt to non-biological velocity profiles, but only when visual feedback was provided.

#### Changes in force-velocity coupling with practice

Experiment 1 demonstrated that the forces applied by humans on the robot were systematically dependent on the deviations of the angular velocity from the biological profile (angular velocity difference). This raised the question whether the attenuation of unwanted forces in the non-biological velocity conditions in Experiment 2 could also be seen in this force-velocity coupling. Does practice weaken this force-velocity coupling or is the observed decrease in forces a more general reduction in forces? To address this issue, the next analysis focused on the subject groups that practiced with feedback in the *constant* and *exaggerated* velocity conditions, which exhibited significant improvements.

**Fig. 11:**
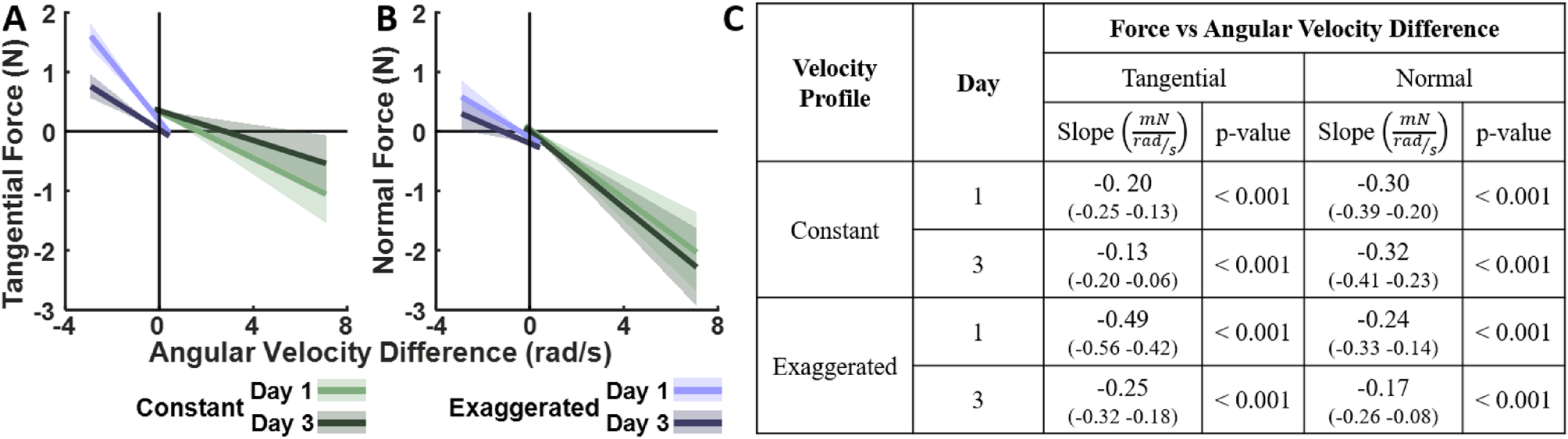
Statistical results for force-velocity coupling in Experiment 2. Regression results on the left include the y-intercepts, mean slopes, and their corresponding 95% confidence intervals for the *constant* and *exaggerated* *with-FB* groups, with the tangential (**A**) and normal (**B**) forces as dependent measures. Velocity profiles and the two days are represented by different colors . The table on the right (**C**) presents the slopes and their corresponding 95% confidence intervals (in parentheses) as well as p-values for post-hoc comparisons of interest, specifically testing whether the slopes differed significantly from zero. The complete table is included in the Supplementary Materials (Table S5).

For this analysis, we examined the continuous signed force profiles of each participant during Day 1 and Day 3. We computed the median force at each spatial point along the ellipse across all 80 trials (320 ellipses) per day, and regressed it against angular velocity difference; velocity condition and day were included as predictors. Figure 11 summarizes the findings. Consistent with Experiment 1, tangential and normal forces showed significant negative slopes with angular velocity difference for both non-biological velocity profiles on Days 1 and 3. While the force-velocity coupling remained significant on Day 3, it weakened with practice in nearly all cases, as seen by the smaller slope values (except for the normal force in the *constant* condition).

Interestingly, while the slopes decreased, the regression intercepts did not significantly differ between the days. This suggests that participants were able to reduce the force-velocity coupling through practice, rather than only lowering the forces throughout. Full statistical results can be found in Table S5 in the Supplementary Material.

## Discussion

With the goal to gain insight into possible barriers for effective physical human–robot interactions, this study focused on identifying human limitations in motor control and in their biomechanics. In two experiments, humans tracked the robot’s end-effector tracing out a horizontal ellipse. Three different velocity-curvature profiles either mimicked the pattern preferred by humans or presented two ‘non-biological’ patterns. The instruction was to not exert any forces against the robot. The robot was feedforward controlled with stiff boundaries to its path. Hence, human forces applied to the robot did not impact the robot trajectory and were ineffective. Unlike in other pHRI studies where the robot and human mutually influenced each other, this asymmetric scenario facilitated to isolate the human’s limitations in such human–robot interactions^44,46^. Grounded in results from human motor control showing that humans modulate their end-effector velocity depending on the trajectories’ curvature, slowing down in curved sections and accelerating in linear segments in a systematic manner, this study demonstrated that human–robot collaboration is smoother and more effective when robots adhere to this velocity-curvature scaling.

Research in motor neuroscience has revealed a number of systematic principles and ‘hard’ limitations in human behavior. Aside from anatomical and biomechanical factors, there are also control limitations. The probably best-known example is the speed-accuracy trade-off^55^. Another ubiquitous control-related feature is the systematic relation of velocity with curvature exhibiting a robust scaling exponent - the ‘one-third power law’. The origin of this relation is still debated: does it reflect neuronal constraints^56^, biomechanical contributions^57^, or does it arise from coupled oscillations^51,58^. Regardless of its origin, the one-third power law has been widely observed across various movements, from hand trajectories to locomotion. Interestingly, the visual system is also sensitive to violations of this law^59^.

Given the ubiquity of this phenomenon, this study examined human adaptation to robot motion that either adhered to or violated the one-third power law. Previous work by our group already provided first indications that robot motion violating this power law elicited undesired interaction forces in humans^34,35^. This study presented a more comprehensive approach, enhancing the experimental design and extending experimental manipulations to address additional new questions. Using the same elliptical tracking task, two experiments attempted to answer the following key questions:

1. Do different non-biological velocity patterns give rise to specific undesired forces resisting the robot’s motion?
2. What role do biomechanical factors play in generating forces when humans track the robot with biological and non-biological velocity profiles?
3. Can extended practice with appropriate feedback mitigate these forces when tracking robot movements?
4. What aspects in the robot trajectory give rise to the undesired force generation?

### Non-biological robot motion elicits unintended forces in human tracking

As already foreshadowed in our previous research, humans cannot follow the robot motion without exerting undesired interaction forces, especially when the robot’s velocity violates the power law.^34^ first reported this, but only in terms of force magnitude. The present study differentiated these observations into the tangential and normal force components which afforded subtler insights, especially when scrutinizing the time-varying forces and learning. Both force components were higher when the robot moved with a non-biological velocity profile, compared to the biological one. Non-zero tangential forces indicated that humans struggled to generate or predict the correct instantaneous speed; non-zero normal forces indicated that they did not predict or follow the direction of motion. Note that the comparison between the two rotational directions did not reveal differences, neither in tangential, nor in normal forces.

Despite the seemingly irreducible forces, even in the biological condition, the applied undesired forces were small, typically around 1-2 N. One might argue that these forces are too low to be detected, especially in blind-folded trials where subjects relied on haptic and proprioceptive information alone. However, humans can detect changes in forces as small as 0.04 N at the wrist, elbow, and shoulder joints, which is important in fine motor skills such as in surgery^60^. Even though these small forces may not fatigue the user, such varying forces may create haptic sensations that deflect attention from the main goals in such joint human–robot tasks.

### Biomechanical factors are secondary

The next question addressed in Experiment 1 was whether human biomechanics shaped these forces. As for example laid out in^61^, the direction of circular crank turning influenced the inertial forces created by a two-joint arm. That experiment strictly constrained the two-joint arm moving in the horizontal plane, which allowed exact calculations of forces generated against the circular constraint. In contrast, our analyses of the RMS forces did not detect major differences in the two rotational directions (except for the normal forces in the constant velocity profile). This indifference to direction may be due to the fact that our study did not constrain human arm movements and participants could choose their preferred configuration in 3D space. Furthermore, in our study the human movements were elliptical with significant changes in curvature and velocity, while in^61^ humans freely moved around a circular channel aiming to maintain constant velocity. Human biomechanics clearly introduces more issues than those manifest in different rotational directions. For example, extension of the human arm is limited by the stationary trunk and stiffness in the shoulder and elbow joint, which present other passive resistive forces. More work is needed to fully explore those biomechanical contributions. Nevertheless, with this caveat, our results suggested that in a leader-follower task, biomechanical factors only played a subordinate role.

### Practice with visual feedback attenuates forces

A critical question for occupational applications is how training influences a participant’s physical interaction with a robot.^34^ had already revealed such improvement in a single 1.5 hour-long experimental session, moving with *constant* velocity. However, this important question on learning needs to be assessed in more than a single session. Not only is one long session fatiguing, thereby underestimating performance improvements, true learning of novel motor skills also requires rest and consolidation^62^. Practice-induced improvements are known to take a long time, not only days, but months and years, and have been modeled with exponentially declining functions^63–65^. The current study tested three daily sessions, each approximately one hour long. Some first results were already reported in^35^ but with limited analyses. As to be expected from other learning curves in sensorimotor skills, the largest improvements were seen on Day 1, followed by continued slower decreases in resistive forces on subsequent days. Though longitudinal studies present challenges, further testing over extended periods would be valuable.

Importantly, these improvements were only seen when subjects received online visual feedback of their applied force in non-biological velocity profiles. The biological profile did not exhibit any changes. When no visual feedback of the applied force was provided, no learning effects were observed. This absence of any improvement, previously shown only for force magnitude^35^, was present in both normal and tangential forces, with the latter showing larger effect sizes. While this was also already foreshadowed in our earlier studies^34^, those previous results require caution as grip force was not controlled and improvements could have been achieved by simply loosening the grip on the handle. In addition, in ^47^ the visual feedback was non-optimal showing a horizontal bar moving vertically around the target level. This vertical orientation of a line cursor was difficult to map onto the horizontal elliptical hand movements, i.e., showed little spatial compatibility. Hence, any improvements may have been compromised by this attention-requiring feedback.

Motivated by this previous experience, the present study aimed to optimize the design to test maximally possible improvement. We developed a spatially compatible display that mimicked the elliptical movements and also used color cues for the circular cursor to indicate too high (red) or acceptable (green) force levels. With this substantial improvement, results unambiguously showed that visual feedback helped human users to decrease their interaction forces over the three days. This suggests that haptic and proprioceptive information about the interaction forces alone was insufficient to fine-tune the users’ interaction with the robot. It also showed that these involuntary forces could not be reduced beyond what is seen in the biological profile.

The design and delivery of augmented feedback is crucial for achieving desired outcomes, and decades of psychological research on stimulus-response compatibility has generated a lot of insights^66^. One example for unhelpful feedback comes from surgical robotics, where novice surgeons received additional haptic information that increased cognitive load and consequently reduced performance accuracy^67^. ^68^ examined object manipulation with a phantom robot and demonstrated that the effectiveness of haptic feedback depends heavily on the task context. These findings highlight that excessive or task-incompatible feedback can hinder human adaptation and negatively impact performance.

### Forces are modulated by angular velocity

Given our robust results from the RMS force values calculated over several ellipses, the next goal was to further specify the determinants for the observed involuntary forces. To begin, the continuous time series of the signed forces exhibited systematic variations within each participant, speaking to their non-random nature. Hence, we examined their modulation across the phases of the ellipse in the three velocity conditions. The most relevant force predictor for the elliptic movement shape was the angular velocity that varied in different ways in the three conditions.

For both experiments, the tangential and normal forces showed systematic dependencies on the angular velocity: within each velocity profile, the larger the angular velocity, the larger the forces. However, the value of the angular velocity alone was not a sufficient predictor of the applied force, since for the same angular velocity, the interaction force differed between velocity conditions. Notably, the directions of increase differed: In the *constant* condition, the tangential forces were negative for higher angular velocities, indicating that humans pulled the robot backward. Conversely, in the *exaggerated* condition, these forces were positive, signaling that humans pushed in direction of motion. A similar behavior was observed for normal forces, with higher angular velocities associated with outward (negative) forces in the *constant* condition, but with inward (positive) forces in the *exaggerated* condition. The *biological* condition also exhibited some systematic increase in both tangential and normal forces with higher robot velocities, though these increases were more moderate than in the non-biological conditions.

Given that the interaction forces were overall smaller in the *biological* condition, and their increase with angular velocity was less, we considered the *biological* profile as a reference and examined how these forces depended on the deviation of angular velocity from the *biological* profile. The results became even more robust. The angular velocity difference significantly predicted the forces exerted, with clear negative slopes and intercepts close to zero in the *constant* and *exaggerated* conditions. Given this finding in Experiment 1, the next question was whether the decreasing forces with practice in Experiment 2 could be seen as a decrease in the slopes of these linear dependencies. Indeed, the force-velocity coupling weakened with practice in nearly all cases, as seen by the smaller slope values on Day 3. This effect was more pronounced for the tangential forces than for the normal forces. Interestingly, while slopes decreased, the regression intercepts did not significantly differ between the days. This suggests that participants were able to reduce the force-velocity coupling through practice, rather than lowering the forces throughout.

**Fig. 12:**
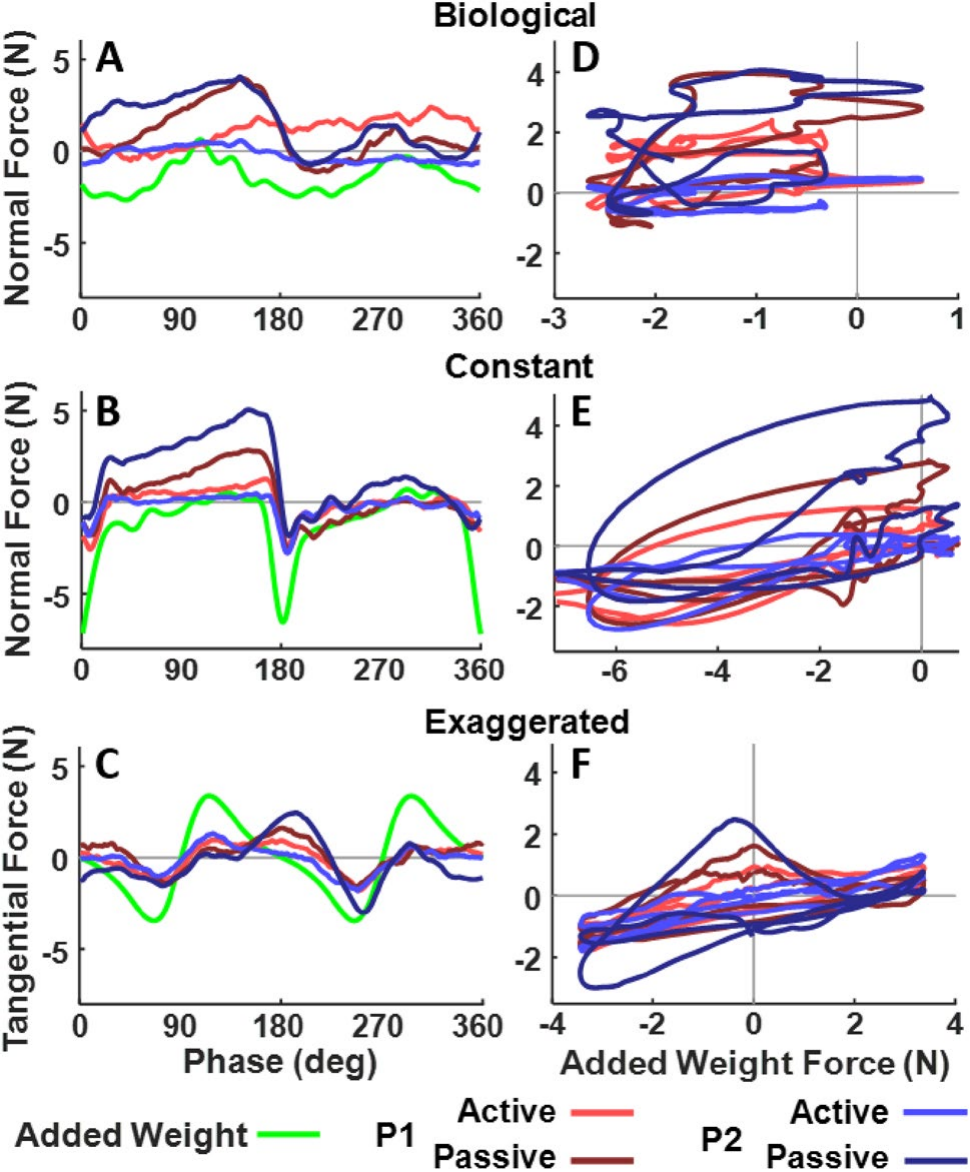
Analysis of the contribution of passive inertial forces to force modulation in human trajectories. **A**, **B**, **C**: Force profiles over phases of the ellipse. **D**, **E**, **F**: Correlation of time series generated by the added weight with the forces generated by two human participants, that were either instructed to remain completely passive or to actively follow the robot motion.

### Inertial forces affect human force control

Given the observed dependency of force on angular velocity, even in the *biological* condition, one can wonder whether the undesired interaction forces were due to inertial forces. To address this issue, we collected additional control trials that added a rigid mass to the robot’s endeffector measuring forces without any interference from human control. Figure 12 A,B,C shows the time series of tangential and normal forces measured in the three velocity conditions. The light green lines denote the forces exerted when a rigid mass of 3 kg was attached to the robot’s endeffector (this weight approximately corresponds to the weight of a human arm). Two human participants were also instructed to perform 12 trials in the three velocity conditions, either actively tracking the robot with zero resistive forces as in the main experiment (lighter shades showing the median trajectories), or letting themselves be dragged passively by the robot (darker shades).

Inspection of the rigid mass profiles reveals negative normal forces, reflecting the centrifugal forces that were higher where angular velocity was higher at the curved portions (at 0 and 180 deg, Fig. 12 A,B). Conversely, tangential forces displayed for the exaggerated condition reached peaks in the two linear portions (90 and 270 deg), where linear velocity was higher (Fig. 12 C). Interestingly, the human modulations of the normal forces around the ellipse were similar in passive humans compared to the rigid mass condition, except the forces were no longer negative. This might be due to the biomechanical constraint of the arm’s attachment to the torso which introduces an additional force (unlike the rigid mass, which was only attached to the robot endeffector). Yet, the modulations typical of inertial forces remained in the passive human traces. When instructed to actively track the robot, humans exerted smaller forces than when remaining passive in all three velocity conditions. This suggested that humans were able to actively resist the passive inertial forces, although incompletely, especially where large inertial forces were at play.

These observations were further illustrated when the human force signals were correlated with the passive inertial forces from the added mass (Fig. 12 D,E,F). The *biological* condition did not exhibit any clear correlation with the inertial forces. This means that participants were largely able to resist the inertial forces in this condition. This general attenuation was similar for the tangential forces (not shown). The *constant* and *exaggerated* conditions presented a different picture: here, the human forces remained correlated with the passive forces, consistent with the stronger force-angular velocity coupling. However, the reduction in force-velocity coupling with practice in the non-biological conditions might suggest that humans learned the specific inertial force patterns and thereby became able to better resist them. While these preliminary results highlight the role of passive inertial forces in shaping human robot tracking, it would be interesting to examine whether with longer training humans could “learn” to counter these inertial forces.

### Further speculations on human control limitations

The observed data may reflect yet other limitations in human motor control. Unlike robot controllers that can regulate force and motion independently in orthogonal directions using hybrid controllers^69–71^, humans are not able to do so^47^, but see^72^. Are the involuntary normal and tangential forces measured in this experiment due to an intermingling of force and motion control? While this is possible in principle, the present task requires humans to generate motion based on their prediction of the robot’s trajectory and execution of a planned trajectory. Hence, the involuntary forces may arise from inadequate prediction of the robot’s trajectory. Relatedly, as discussed above, the inertial forces that naturally arise in curved motion may be insufficiently sensed and learned. If this were the case, the forces deviating from the robot path could also be due to prediction errors. While the results of our study shed some light on human limitations, further experimentation is needed to better understand this behavior.

## Conclusions for robotic applications

These results have important implications for human–robot system design and deployment in rehabilitation, collaborative manufacturing, and design of supernumerary limbs, where smooth and synergetic human–robot interaction is critical^13,73,74^. While robots can be programmed to follow almost any desired trajectory, human physiological and neural constraints pose challenges or even hard limitations for effective collaboration. Hence, to foster intuitive and synergistic interactions, the design and control strategies of robots need to take human factors into consideration. When deploying a human–robot system in which the robot has to move with a non-biological pattern, due to task or environmental constraints, dedicated visual feedback of the performance should be provided, at least during a training period when the system is introduced. This seems necessary to allow or facilitate learning the robot’s motion pattern, which is a crucial factor to improve acceptance of the system and ensure its actual use and usefulness. If augmented visual feedback cannot be provided for various reasons (e.g., technical, cost), the robot should preferably be designed to move according to a biological pattern, which offers the best performance in the absence of augmented feedback.

## Supplementary Information

Supplementary Information 1.

Supplementary Information 2.

## Data Availability

Data sets generated during the current study are available from the corresponding author on reasonable request.
