# Supplementary material for: Force-velocity coupling limits human adaptation in physical human–robot interaction
Source: Sci Rep. 2026 Jan 16;16:5427. doi: 10.1038/s41598-025-34959-4 (PMC12887034; doi:10.1038/s41598-025-34959-4)
Supplement: Supplementary file 1 — Supplementary Information 1. [file 41598_2025_34959_MOESM1_ESM.pdf]

Experiment 1: Performance in Different Velocity Profiles and Directions

Table S1. Results of the linear mixed model analysis (Omnibus test and pairwise post-hoc comparisons) examining the effects of velocity profile and direction on overall force magnitude and its components tangential and normal force in Experiment 1. Degrees of freedom are represented by the acronym df. Chi-squared (χ^2) statistics from the Omnibus tests quantify deviations from the null model, and the associated p-values indicate the probability of observing such deviations if no effect is present.

			**Force**								
			Magnitude			Tangential			Normal		
**Omnibus Test**				χ^2	p		χ^2	p		χ^2	p
	Velocity* Direction<br>df between nested models: 2			0.11	0.74		0.08	0.78		0.2	0.65
				54.9	**1e-4**		19.2	**1e-3**		83.2	**1e-4**
**Post-hoc Comparisons**	df: 1270		β	t	p	β	t	p	β	t	p
	Biological/Constant		0.78	-6.09	**1e-4**	0.79	-4.71	**1e-4**	0.8	-4.35	**1e-4**
	Biological/Exaggerated		0.83	-4.47	**1e-4**	0.73	-6.28	**1e-4**	0.94	-1.23	0.44
	CCW/CW		1.03	0.67	0.5	1.02	0.34	0.73	1.05	0.8	0.42
	CW	Biological/Constant	0.83	-4.2	**1e-4**	0.81	-3.9	**1e-4**	0.88	-2.45	**0.03**
	CW	Biological/Exaggerated	0.8	-4.94	**1e-4**	0.71	-6.61	**1e-4**	0.9	-1.89	0.11
	CCW	Biological/Constant	0.74	-6.65	**1e-4**	0.77	-5.18	**1e-4**	0.73	-5.85	**1e-4**
	CCW	Biological/Exaggerated	0.86	-3. 5	**1e-3**	0.75	-5.56	**1e-4**	0.98	-0.44	0.9
	CCW/CW	Biological	1.02	0.34	0.74	1.01	0.29	0.77	1.01	0.22	0.83
	CCW/CW	Constant	1.13	2.6	**0.01**	1.07	1.46	0.14	1.22	3.14	**2e-3**
	CCW/CW	Exaggerated	0.95	-0.98	0.32	0.96	-0.84	0.4	0.94	-1.02	0.31

Experiment 1: Force and Motion Coupling - Angular Velocity

Table S2. Results of linear mixed model analysis examining the linear relation between the human applied force and angular velocity in Experiment 1. The degrees of freedom (df) are the same throughout the omnibus test and the post-hoc comparisons. Chi-squared (χ^2) statistics from the Omnibus tests quantify deviations from the null model, and the associated p-values indicate the probability of observing such deviations if no effect is present.

			**Force**			
			Tangential		Normal	
**Omnibus Test**	df between nested models: 2		χ^2	p	χ^2	p
	Velocity Profile* Angular Velocity* Direction		17.9	**1e-4**	32.36	**1e-4**
**Post-hoc Comparisons**	df: 40471					
	Velocity Profile	Direction	t	p	t	p
	Biological	CW	6.9	**1e-4**	5.6	**1e-4**
		CCW	9.57	**1e-4**	2.6	0.06
	Constant	CW	-3.51	**0.003**	-4.38	**1e-4**
		CCW	-2.67	0.04	-5.76	**1e-4**
	Exaggerated	CW	26.04	**1e-4**	17.4	**1e-4**
		CCW	29.05	**1e-4**	10.08	**1e-4**

Experiment 1: Force and Motion Coupling - Angular Velocity Difference

Table S3. Results of linear mixed model analysis examining linear relation between human applied force and angular velocity difference in experiment 1. The degrees of freedom (df) are the same throughout the omnibus test, and the post-hoc comparisons. Chi-squared (χ^2) statistics from the Omnibus tests quantify deviations from the null model, and the associated p-values indicate the probability of observing such deviations if no effect is present.

			Force			
			Tangential		Normal	
**Omnibus Test**	df between nested models:2		χ^2	p	χ^2	p
	Velocity Profile* Angular Velocity* Direction		26.28	**1e-4**	54.39	**1e-4**
**Post-hoc Comparisons**	df: 26975					
	Velocity Profile	Direction	t	p	t	p
	Constant	CW	-4.76	**1e-4**	-9.0	**1e-4**
		CCW	-3.66	**1e-3**	-11.97	**1e-4**
	Exaggerated	CW	-14.4	**1e-4**	-16.03	**1e-4**
		CCW	-16.41	**1e-4**	-10.29	**1e-4**

Experiment 2: Performance in Different Velocity Profiles and Feedback

Table S4. Results of linear mixed model analysis (Omnibus test) examining the effects of velocity profile and direction on overall force magnitude and its components tangential and normal force in Experiment 1. The post-hoc comparisons had 9388 degrees of freedom (df). Chi-squared (χ^2) statistics from the Omnibus tests quantify deviations from the null model, and the p-values indicate the probability of observing such deviations if no effect is present.

				Force					
				Tangential			Normal		
Omnibus Test	Velocity Profile* Feedback* Day* Trial		df between nested models: 4		χ^2	p		χ^2	p
					48.3	**1e-4**		17.57	**1e-3**
	Ratio	Feedback	Velocity Profile	β	t	p	β	t	p
	$Day\ 1/Day\ 3$	No-FB	Biological	0.86	-1.8	0.17	0.96	-0.36	0.93
			Constant	1.1	1.12	0.5	1.05	0.5	0.87
			Exaggerated	0.9	-1.3	0.4	0.92	-1	0.58
		With-FB	Biological	1.08	1.03	0.56	1.03	0.34	0.94
			Constant	1.45	4.72	**1e-4**	1.25	2.6	**0.02**
			Exaggerated	1.42	4.5	**1e-4**	1.33	3.33	**0.003**
	Slope over Day 1 Trials	With FB	Biological	-0.003	-2.82	**0.01**	-0.0023	-1.9	0.16
			Constant	-0.004	-3.58	**0.001**	-0.0043	-3.54	**0.0012**
			Exaggerated	-0.004	-3.38	**0.002**	-0.004	-3.29	0.14
		No-FB	Biological	0.004	2.9	**0.01**	0.0052	3.65	**0.0008**
			Constant	0.0005	0.5	0.94	-0.0001	-0.1	0.99
			Exaggerated	0.002	1.81	0.19	0.0023	1.77	0.21
	Slope over Day 2 Trials	With FB	Biological	-0.001	-1.3	0.45	-0.0011	-0.9	0.76
			Constant	-0.0006	-0.59	0.91	-0.001	-0.85	0.78
			Exaggerated	-0.001	-1.28	0.49	-0.002	-1.96	0.14
		No-FB	Biological	0.004	3.34	**0.002**	0.0028	1.96	0.14
			Constant	0.0004	0.37	0.98	-0.0006	-0.52	0.94
			Exaggerated	0.003	3.07	**0.006**	0.004	2.99	**0.008**
	Slope over Day 3 Trials	With FB	Biological	0.0006	0.62	0.9	-0.0005	-0.48	0.95
			Constant	-0.001	-1.47	0.37	-0.0008	-0.68	0.87
			Exaggerated	-0.0007	-0.7	0.49	-0.0001	-0.1	0.99
		No-FB	Biological	0.001	0.9	0.75	0.001	0.69	0.86
			Constant	0.0012	1.08	0.63	0.0001	0.12	0.99
			Exaggerated	-0.001	-1.04	0.65	0.002	1.28	0.49

Experiment 2: Force and Motion Coupling - Angular Velocity Difference

Table S5. Results of the linear mixed model analysis (Omnibus test and pairwise post-hoc comparisons) examining linear relation between human applied force and angular velocity difference in Experiment 2. The degrees of freedom (df) are the same throughout the omnibus test, and the post-hoc comparisons. Chi-squared (χ^2) statistics from the Omnibus tests quantify deviations from the null model, and the associated p-values indicate the probability of observing such deviations if no effect is present.

			**Force**			
			Tangential		Normal	
**Omnibus Test**	df between nested models: 1		χ^2	p	χ^2	p
	Velocity Profile* Angular Velocity* Day		53.99	**1e-4**	24.84	**1e-4**
**Post-hoc Comparisons**	df: 10480					
	Velocity Profile	Day	t	p	t	p
	Constant	1	-6.99	**1e-4**	-7.77	**1e-4**
		3	-4.7	**1e-3**	-8.72	**1e-4**
		Slope difference between Day 1 and Day 3	-4.82	**1e-4**	2.77	**0.006**
		Intercept difference between Day and Day 3	-0.1	0.92	0.036	0.7
	Exaggerated	1	-16.87	**1e-4**	-6.08	**1e-4**
		3	-9.08	**1e-4**	-4.57	**1e-4**
		Slope difference between Day 1 and Day 3	-13.33	**1e-4**	-4.14	**1e-4**
		Intercept difference between Day and Day 3	0.84	0.4	0.095	0.27
